# Cell-Free DNA–Derived Immune Cell Ratios Uncover Cancer-Associated Systemic Changes

**DOI:** 10.1158/2767-9764.CRC-25-0747

**Published:** 2026-04-13

**Authors:** Laura Andersen, Sia V. Lindskrog, Iver Nordentoft, Amanda Frydendahl, Jesper Nors, Tenna V. Henriksen, Mads H. Rasmussen, Lene H. Iversen, Kåre A. Gotschalck, Jørgen B. Jensen, Lars Dyrskjøt, Claus L. Andersen, Nicolai J. Birkbak

**Affiliations:** 1 Department of Molecular Medicine, Aarhus University Hospital, Aarhus, Denmark.; 2 Department of Clinical Medicine, Aarhus University, Aarhus, Denmark.; 3 Bioinformatics Research Center, Aarhus University, Aarhus, Denmark.; 4 Department of Clinical Epidemiology, Aarhus University, Aarhus, Denmark.; 5 Department of Surgical Gastroenterology, Aalborg University Hospital, Aalborg, Denmark.; 6 Department of Clinical Medicine, Aalborg University, Aalborg, Denmark.; 7 Department of Surgery, Horsens Regional Hospital, Horsens, Denmark.

## Abstract

Genome-wide coverage patterns of plasma cell-free DNA (cfDNA) fragments reflect nucleosome positioning in the cells of origin, enabling noninvasive inference of cell type contributions and transcriptional activity. Whereas the majority of cfDNA originates from hematopoietic cells, the diagnostic and biological relevance of this fraction remains underexplored. In this study, we performed cfDNA-based deconvolution of blood cell types by integrating transcription start site (TSS) coverage profiles from plasma whole-genome sequencing with single-cell transcriptomic reference data. By correlating cfDNA TSS coverage with gene expression across 457 blood cell types, we ranked their relative contributions to the cfDNA pool. We analyzed 788 pretreatment and longitudinal plasma samples from patients with localized colorectal cancer, muscle-invasive bladder cancer (MIBC), as well as 30 samples from healthy controls. In healthy individuals, cfDNA TSS coverage profiles reflected blood gene expression, and the inferred cell type contributions recapitulated the known hematopoietic composition. In patients with cancer, we observed a significant increase in cfDNA contributions from lymphocytes, including T cells and plasma cells, and decreased contributions from monocytes and granulocytes. These immune-derived signatures distinguished patients with colorectal cancer (AUC = 0.793) and MIBC (AUC = 0.745) from healthy controls. Longitudinal analysis of immune cell type contributions revealed treatment-associated changes in the relative abundance of classic monocytes and plasma cells, although these temporal dynamics were not predictive of relapse or outcome. Together, these findings suggest that cfDNA-derived immune signatures may capture aspects of systemic immune remodeling in cancer, potentially providing a complementary noninvasive biomarker in liquid biopsies beyond tumor-derived signals.

**Significance:**

There is limited understanding of how plasma cfDNA reflects systemic immune changes in cancer. We show that cfDNA coverage profiles reflect blood cell gene expression and can be used to infer blood cell ranks that recapitulate known cell compositions. These cfDNA-derived ranks reveal cancer-associated immune alterations that distinguish patients with cancer from healthy controls. Our findings highlight cfDNA as a minimally invasive biomarker of systemic immune remodeling in cancer.

## Introduction

Plasma cell-free DNA (cfDNA) consists of short DNA fragments released into the bloodstream predominantly through apoptosis and necrosis (1). In healthy individuals, the majority of cfDNA is of hematopoietic origin, reflecting the dynamics and abundance of white blood cell populations (2, 3). In patients with cancer, additional cfDNA is shed from tumor cells—termed circulating tumor DNA (ctDNA)—which carries somatic mutations and other tumor-specific characteristics. ctDNA has shown great clinical potential as a noninvasive biomarker for early cancer detection, prognosis assessment, and treatment monitoring, as extensively reviewed by Bruhm and colleagues (4) and Bartolomucci and colleagues (5). However, the cell type composition and potential clinical relevance of the hematopoietic-derived cfDNA fraction remains underexplored.

The fragmentation patterns of cfDNA reflect the chromatin landscape of the originating cells and have been shown to correlate with gene expression (6, 7). Actively transcribed genes are characterized by being nucleosome-depleted at transcription start sites (TSS), leaving these sites more susceptible to nuclease-mediated breakdown during apoptosis and while in the circulation. Consequently, TSSs of expressed genes have reduced cfDNA coverage, whereas coverage remains high at TSSs of inactive genes. This phenomenon enables noninvasive inference of transcriptional programs in hematopoietic and cancer cells contributing to plasma cfDNA (6–8).

Leveraging these fragmentomic features, cfDNA-based cell type deconvolution methods have emerged to characterize tissue and cell type contributions to the cfDNA pool. A recent study by Stanley and colleagues (9) correlated single-cell transcriptomic profiles with cfDNA coverage patterns from breast cancer, colorectal cancer, and multiple myeloma. They identified tissue-resident immune cell types distinguishing patients with cancer from healthy individuals. Similarly, Mathios and colleagues (10) showed that cfDNA fragmentomic profiles can be used to classify brain cancers. These profiles captured not only tumor-derived signals but also contributions from white blood cells, underscoring the relevance of immune cell signatures in cancer detection.

These findings suggest that immune cell contributions to the cfDNA pool carry diagnostic and biological relevance. We therefore hypothesized that immune-derived cfDNA fragmentation patterns may provide a noninvasive approach to profile the immune composition. Such profiles may be used to search for differences between healthy and diseased individuals and may also be used to explore how the immune composition changes over time, e.g., in response to therapy. To investigate this, we applied a cfDNA-based cell type deconvolution strategy based on cfDNA coverage profiles at TSSs of protein-coding genes. We analyzed plasma whole-genome sequencing (WGS) data from previously described cohorts of patients with locally advanced colorectal cancer, muscle-invasive bladder cancer (MIBC), and healthy individuals (Table 1; refs. 11, 12). We first demonstrated how TSS coverage reflects blood cell gene expression in healthy individuals. We then applied blood cell type deconvolution to identify differential immune cell signatures between cancer and control groups. Finally, we tracked longitudinal immune cell dynamics in cfDNA throughout treatment. These findings indicate that cfDNA may provide insights into systemic immunologic changes in health and disease.

**Table 1.** Clinical characteristics of the study cohorts.

Characteristic	CRC *N* = 124^a^	MIBC *N* = 102^a^	Healthy control *N* = 30^a^
Sex	​	​	​
Female	54 (44%)	20 (21%)	16 (53%)
Male	70 (56%)	77 (79%)	14 (47%)
Age	66 (59, 72)	68 (62, 72)	66 (60, 73)
OS months	44 (27, 56)	53 (35, 72)	—
Relapse	​	​	​
Nonrelapse	91 (73%)	73 (78%)	—
Relapse	33 (27%)	21 (22%)	—
Dead	13 (10%)	23 (24%)	—
MSI status	​	​	​
MSI	20 (16%)	—	—
MSS	104 (84%)	—	—
Smoking status	​	​	​
Current	—	42 (43%)	—
Former	—	39 (40%)	—
Never	—	15 (15%)	—
Unknown	—	1 (1%)	—

Summary of baseline clinical and demographic features for patients with CRC, MIBC, and healthy controls included in this study. Characteristics include age, sex, OS (reported in months), recurrence, MSI/MSS status, and smoking status.

Abbreviations: CRC, colorectal cancer; MSI, microsatellite instable; MSS, microsatellite stable; OS, overall survival.

a *n* (%); Median (Q1, Q3).

## Materials and Methods

### Patient participants

Patients with colorectal cancer and MIBC and noncancer controls included in this study were previously described in Frydendahl and colleagues (11) and Nordentoft and colleagues (12). Briefly, the study cohort comprised 124 patients with Union for International Cancer Control stage III colorectal cancer and 102 patients with localized MIBC, all treated according to national clinical guidelines. Patients with colorectal cancer underwent surgical resection with curative intent followed by adjuvant chemotherapy (ACT). These patients had blood samples collected preoperatively (pre-op), postoperatively (post-op), and every 3 months for up to 3 years. Patients with MIBC received neoadjuvant chemotherapy (NAC) followed by radical cystectomy (RC) and had blood samples collected before treatment, during NAC, pre-op, post-op, and throughout follow-up for up to 2 years. Additionally, 30 noncancer controls were included from the Colorectal Cancer Research Biobank at Aarhus University Hospital. The clinical characteristics of the study cohorts, including sex, age, and survival information, are provided in Table 1.

### Ethics approval and consent to participate

The study was performed in accordance with the Declaration of Helsinki, and all participants provided written informed consent. The study was approved by the National Committee on Health Research Ethics (#1302183 and #1706291) and the Committees on Biomedical Research Ethics in the Central Region of Denmark (1–16-02–453-14 and 1–10-72–3-18).

### Plasma sample collection, cfDNA processing, and sequencing

A detailed description of the methodology can be found in Frydendahl and colleagues (11) and Nordentoft and colleagues (12). Overall, cfDNA was extracted from plasma using the QiaAMP Circulating DNA Kit (Qiagen) and assessed for leukocyte contamination. Libraries were prepared from 2 mL of plasma using xGen UDI-AMI Adapters (IDT) and KAPA HyperPrep Kit (Roche), with post-ligation and post-PCR cleanups performed using AMPURE beads at 1.4× and 1× (beads/DNA) ratios, respectively. Libraries were amplified over seven PCR cycles, quantified using the Qubit dsDNA BR Assay Kit (Thermo Fisher Scientific), and assessed for size distribution using the TapeStation D1000 (Agilent). Germline DNA libraries were prepared using the Twist Library Preparation EF Kit (Twist Bioscience). Germline DNA and cfDNA samples were subjected to WGS on the NovaSeq platform (Illumina) using paired end-sequencing (2 × 150 base pairs) to a target coverage of 20×. Demultiplexing was performed based on predefined indices, and raw reads were converted to FASTQ format using the illumina tool “bcl2fastq” (RRID: SCR_015058).

### Preprocessing of WGS data and somatic mutation calling

FASTQ files were adapter-trimmed (Skewer v0.2.2; RRID: SCR_001151) and aligned to GRCh38 (Burrows-Wheeler Aligner MEM v0.7.17; RRID: SCR_010910). BAM files were sorted and indexed (Samtools v 2.14; RRID: SCR_002105), marked for duplicate reads (GATKMarkDuplicatesSpark v 4.1.8.0; RRID: SCR_001876), and evaluated for per-read base quality score (GATK BQSRPipelineSpark; RRID: SCR_001876). Alignment quality control metrics were calculated using Picard (RRID: SCR_006525) and GATK (RRID: SCR_001876). Somatic single-nucleotide variants were called using GATK Mutect2 (v4.2.4.1; RRID: SCR_026692) and Strelka2 (v2.9.10; RRID: SCR_005109), retaining only consensus PASS variants (GATK FilterMetectCalls) not found in dbSNP (RRID: SCR_002338) or cfDNA from healthy individuals. INDELs were called with SVaba (v1.1.3; RRID: SCR_022998) and Mutect2 (v4.2.4.1; RRID: SCR_026692), keeping overlapping calls.

### ctDNA detection

ctDNA detection and tumor fraction (TF) estimation was performed using a proprietary bioinformatic pipeline based on a previously described method (13), which integrates an error-suppression model derived from WGS of healthy cfDNA and patient-specific mutation profiles.

### Single-cell transcriptomic reference dataset

Single-cell RNA-sequencing data were obtained from the Tabula Sapiens reference dataset (14) via CZ CellxGENE Discover. Cell types provided in the atlas published in 2022 were used, comprising 457 annotated cell types. The Tabula Sapiens dataset comprises cells sampled across multiple tissues, allowing individual cells to be represented in more than one tissue context and providing a comprehensive reference of cell type–specific transcriptional programs across the human body. Cell type gene expression was averaged across cells within each cell type to generate cell type–specific expression profiles. Cell type categories were defined according to Supplementary Table S1 into monocytes, lymphocytes, granulocytes, progenitor and erythroid lineage, endothelial cells, other tissue cells, stromal cells, and hepatic lineage. Additional grouping into compartments and tissues was based on Tabula Sapiens annotations.

### TSS coverage and cell-of-origin analysis

The mean GC-corrected coverage at ±1,000 base pairs surrounding the TSS of protein-coding genes (RefSeq database; RRID: SCR_003496) was calculated using Griffin (15), following the Griffin wiki (https://github.com/adoebley/Griffin, last accessed January 29, 2026). Multiple transcripts per gene were aggregated by computing the mean. Cell type ranking was performed using the 16,080 protein-coding genes shared between RefSeq and the Tabula Sapiens single-cell reference dataset. For each cell type, gene expression profiles were correlated with cfDNA TSS coverage using Spearman correlation. Cell types were then ranked by the strength of the negative correlation, with stronger negative correlations indicating greater inferred cfDNA contribution.

### Bulk RNA-sequencing reference data

Transcripts per million (TPM) gene expression data were obtained from the Genotype-Tissue Expression (GTEx) project via the GTEx Portal (https://gtexportal.org, last accessed January 29, 2026). We downloaded the file gene_tpm_v10_whole_blood.gct.gz, which contains normalized gene-level TPM values from GTEx whole blood samples. For each gene, the mean TPM across all samples was calculated to generate a bulk gene expression profile representative of blood-derived RNA. Genes with a mean TPM of 0 defined as unexpressed, and the top 2,000 genes by TPM were identified for comparison with TSS coverage estimates.

### ImmuneLENS

T-cell fractions were estimated using ImmuneLens (16), a bioinformatic pipeline for inferring T-cell content from WGS data. The method estimates the T-cell abundance based on read coverage T cell–specific genomic loci. We applied immuneLENS to germline WGS data following the guidelines provided in the ImmuneLENS documentation (https://github.com/McGranahanLab/ImmuneLENS). The resulting T-cell fraction is reported as the percentage of total DNA in the sample that is T cell–derived.

### Statistical analysis

All analyses were performed using R (v4.4.1), and statistical significance was defined as *P* value < 0.05. Cell types were grouped into broader categories by calculating the median rank within each group. Comparison of cell type ranks between case–control groups or across time points (e.g., baseline vs. treatment) were performed using two-sided Wilcox rank-sum tests. Correlation between cell type ranks and T-cell fractions were assessed using Spearman’s correlation. *P* values were adjusted for multiple testing using the false discovery rate method. Fold changes (FC) were computed as the ratio of median ranks in cancer versus control groups. Data processing and visualization were carried out using the *tidyverse* (v2.0.0; RRID: SCR_019186), *ggplot2* (v3.5.1; RRID: SCR_014601), *stats* (v4.4.1; RRID: SCR_025678), and *ggpubr* (v0.6.0; RRID: SCR_021139) packages.

For the classification analysis, we trained LASSO-regularized logistic regression models implemented using the *caret* package (v7.0.1; RRID: SCR_021138). For cancer classification, separate models were trained for colorectal cancer and MIBC using cfDNA-derived cell type ranks of monocytes, lymphocytes, and granulocytes across all tissues to distinguish patients with cancer from healthy controls. For relapse prediction, separate models were trained in each cohort to classify relapse versus nonrelapse using the top 10 immune cell types (monocytes, lymphocytes, and granulocytes) showing the highest interquartile range (IQR) in rank change from the pretreatment sample to the first chemotherapy time point (day 90 of ACT for colorectal cancer; day 20 of NAC for MIBC). Hyperparameter tuning was performed over a grid of 100 log-spaced λ values (10^−4^ to 10^1^) using the *glmnet* method with standardized predictors. Model performance was evaluated using leave-one-out cross-validation (LOOCV), with AUC as the performance metric. Final model performance was assessed using the *pROC* package (v1.18.5; RRID: SCR_024286). For cancer classification, immune model detection was defined using a 95% specificity threshold.

Survival analysis was performed based on the predicted relapse probabilities, with patients stratified into “high” and “low” immune score groups using the median predicted probability as a threshold. Kaplan–Meier survival curves were generated for recurrence-free survival (RFS), with follow-up censored at 3 years, using the *survival* (v3.8.3; RRID: SCR_021137) and *survminer* (v0.5.0; RRID: SCR_021094) packages.

## Results

### Coverage at TSSs reflects gene expression in hematopoietic cells

**Figure 1.**
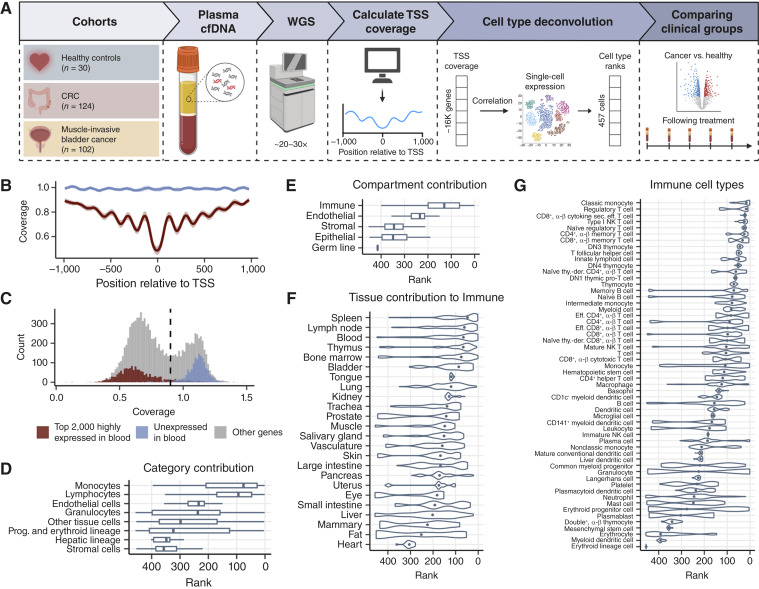
cfDNA coverage at TSSs enables deconvolution of blood cell type contributions. **A,** Schematic overview of the analytic approach. Plasma cfDNA is subjected to WGS at a target depth of 20–30×. GC-corrected coverage at TSS of protein-coding genes is computed using Griffin (15). TSS coverage is then correlated with gene expression profiles from the Tabula Sapiens single-cell reference dataset (14) to generate cell type ranks, reflecting relative contributions to the cfDNA pool. **B,** Mean cfDNA coverage in healthy individuals across ±1,000 base pairs surrounding the TSS of the top 2,000 highly expressed (red) and unexpressed (blue) genes in blood. Expression categories were defined using the GTEx dataset, with highly expressed genes ranked by TPM and unexpressed genes defined as those with TPM = 0. **C,** Distribution of median TSS coverage for all protein-coding genes in healthy individuals, overlaid with gene expression categories from GTEx: unexpressed (blue), expressed (red), and all other genes (gray). **D,** Inferred cell type ranks in healthy individuals grouped by cell type categories in healthy controls. Higher ranks indicate greater relative cfDNA contribution. Prog., progenitor. **E,** Same as **D**, grouped by cell type compartment defined by Tabula Sapiens. **F,** Cell types contributing to the Immune compartment grouped by tissue. **G,** Cell type ranks for individual immune cell types. CRC, colorectal cancer; eff., effector; NK, natural killer; Sec., secreting; thy.-der., thymus-derived.

To assess whether cfDNA coverage at TSSs could reliably capture gene expression profiles in blood cells, we applied Griffin, a computational framework for analyzing nucleosome positioning and GC-corrected cfDNA coverage from plasma WGS data (15). In plasma WGS samples from healthy individuals (*n* = 30), we quantified the mean cfDNA coverage at the promoter region (±1,000 base pairs surrounding the TSS) of protein-coding genes annotated in RefSeq (Fig. 1A; ref. 17).

To first validate that cfDNA coverage at TSSs reflects the underlying gene activity of blood cells, we compared TSS coverage with gene expression profiles from the GTEx whole blood dataset (18). Unexpressed genes were defined as those with TPM equal to zero, whereas highly expressed genes were defined as the top 2,000 genes ranked by TPM. Consistent with previous findings (6, 7), highly expressed genes showed a characteristic depletion of cfDNA coverage at the promoter region surrounding the TSS, indicative of nucleosome-depleted regions, whereas unexpressed genes showed a uniform, high-coverage profile (Fig. 1B). Furthermore, evaluating the mean TSS coverage per gene displayed a bimodal distribution, with the two modes corresponding to the gene expression status in blood cells (Fig. 1C). Specifically, unexpressed genes exhibited high TSS coverage, whereas highly expressed genes showed low TSS coverage. Together, these results confirm that cfDNA fragmentation patterns are strongly associated with gene activity in blood-derived cells.

### cfDNA coverage at TSSs enables hematopoietic cell type inference

As transcriptional programs are highly cell type–specific, we next thought to investigate whether cfDNA TSS coverage profiles could be used to deconvolute the cellular origins of cfDNA. To this end, we correlated the TSS coverage profiles with average gene expression signatures from 457 annotated cell types in the Tabula Sapiens single-cell transcriptomic atlas (Fig. 1A; ref. 14). As cfDNA coverage around TSSs inversely reflects gene expression, stronger negative correlations between TSS coverage and gene expression indicate greater cfDNA contribution from the corresponding cell type. Based on this principle, cell types were ranked according to the strength of their negative correlation with cfDNA TSS coverage, with higher-ranking cell types inferred to contribute more cfDNA to the plasma.

As a benchmark for our cfDNA-based cell type inference, we applied the method to plasma samples from healthy individuals (*n* = 30). Consistent with prior reports (2, 3, 9), the predominant contributors to the cfDNA pool were monocytes, lymphocytes, endothelial cells, and granulocytes (Fig. 1D). Evaluating cell type compartment as defined by Tabula Sapiens, immune cells revealed the strongest correlation with cfDNA TSS coverage (Fig. 1E). In contrast, stromal and epithelial cell types were predominantly ranked below 200, in line with their minimal contribution to cfDNA under healthy conditions and serving as a negative control. We next grouped cell types in each compartment by tissue origin. Within the immune compartment, the spleen, lymph node, blood, and thymus were ranked among the highest contributing tissues (Fig. 1F), consistent with their known roles in hematopoiesis and immune cell production. In addition, evaluating the nonimmune compartments revealed cell type ranks below 200 for the majority of tissues, further supporting their limited contribution (Supplementary Fig. S1). Finally, we stratified the immune signal by individual cell types and found the strongest contributions from monocyte subsets, natural killer T cells, CD8^+^ T cells, and CD4^+^ T cells (Fig. 1G). Together, these findings demonstrate that cfDNA TSS coverage profiles can be used to deconvolute hematopoietic cell type contributions, producing cell type ranks that align with the known composition of cfDNA under healthy physiologic conditions.

### Adaptive immune cell–derived cfDNA is enriched in patients with cancer

Previous studies have shown that the cellular contributions to the cfDNA pool reflect underlying biological processes in cancer, including alterations in immune cell turnover (9, 10). Based on these findings, we hypothesized that cancer-associated changes in blood cell composition would be detectable through cfDNA-derived cell type profiles. To investigate this, we applied our cell type deconvolution approach to pretreatment plasma samples from patients with colorectal cancer (*n* = 124; ref. 11) and MIBC (*n* = 102; ref. 12), as well as healthy controls (*n* = 30). These cancer cohorts had ctDNA detection rates of 83% in colorectal cancer and 46% in MIBC (Supplementary Fig. S2A). TFs among ctDNA-positive samples were low (IQR: 0.056%–0.64% and 0.04%–0.52%, respectively; Supplementary Fig. S2B), reflecting the challenging low–tumor burden setting in these patients.

**Figure 2.**
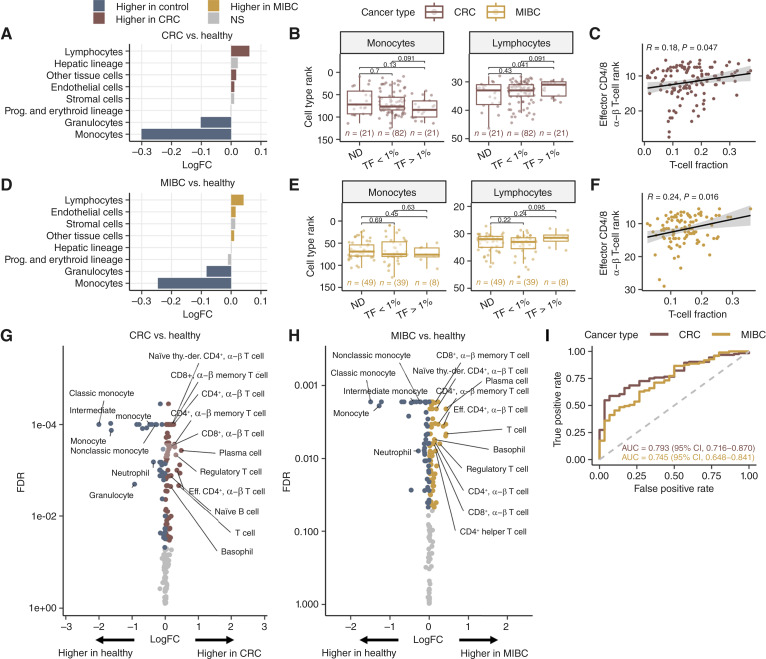
Cancer-associated shifts in immune cell contributions to cfDNA. **A,** Log_2_ FC (logFC) in cell type ranks grouped by category between healthy individuals (blue, *n* = 30) and patients with colorectal cancer (CRC; red, *n* = 124). Higher ranks reflect greater inferred cfDNA contribution. **B,** Comparison of median cell type ranks for monocytes (left) and lymphocytes (right) from the spleen, lymph node, blood, and thymus across ctDNA TF categories in CRC: ctDNA ND, ctDNA TF <1%, and TF >1%. *P* values were calculated using two-sided Wilcoxon rank-sum tests. **C,** Correlation between cfDNA-derived ranks for effector CD4^+^ and CD8^+^ α–β T cells from lymph node and blood and T-cell fraction estimated using ImuneLENS (16) from germline WGS. Spearman’s correlation coefficient (r) and *P* value are shown. **D,** Same as in **A**, comparing healthy individuals (blue, *n* = 30) and patients with MIBC (yellow, *n* = 102). **E,** Same as **B**, for MIBC. **F,** Same as **C**, for MIBC. **G,** Volcano plot showing logFC in cfDNA-derived ranks vs. false discovery rate (FDR)-adjusted *P* values for individual immune cell types (monocytes, lymphocytes, and granulocytes) between healthy individuals (blue) and patients with CRC (red). Cell types with the highest FC are labeled. For cell types present in multiple tissues, only the instance with the highest log FC is shown. *P* values were calculated using two-sided Wilcoxon rank-sum tests and adjusted for multiple testing using the FDR method. **H,** Same as **G**, comparing healthy individuals and patients with MIBC (yellow). **I,** Receiver operating characteristic (ROC) curves for LASSO-regularized logistic regression models classifying CRC (red) and MIBC (yellow) vs. healthy controls. Models were trained on cell type ranks from monocytes, lymphocytes, and granulocytes, and evaluated using LOOCV. Area under the curve (AUC) and 95% CIs are indicated. Eff., effector; NS, not significant; Prog., progenitor; thy.-der., thymus-derived.

We first compared broad cell type categories between patients with cancer and healthy controls. In the colorectal cancer cohort, lymphocyte-derived cfDNA contributions were significantly increased, whereas monocyte-derived cfDNA contributions were decreased relative to healthy controls (Fig. 2A). To investigate whether these shifts were associated with pretreatment plasma tumor burden, we analyzed lymphocyte and monocyte ranks derived from blood-related tissues (spleen, lymph node, blood, and thymus) across ctDNA TF groups: ctDNA not detected (ND), TF <1%, and TF >1%. Lymphocyte-derived cfDNA revealed a significant increase in samples with TF >1% compared with ctDNA ND patients (*P* = 0.041), whereas monocyte ranks showed a modest but nonsignificant decrease (*P* = 0.091; Fig. 2B). Further stratification of the TF <1% group into TF <0.1% and 0.1% < TF < 1% revealed no differences between subgroups (Supplementary Fig. S3A), suggesting that immune-derived cfDNA shifts are primarily detectable in patients with higher plasma tumor burden and are less discernible at TFs below 1%. As a final validation of the biological relevance of our lymphocyte signal, we compared cfDNA-derived ranks to an orthogonal estimate of T-cell abundance derived from germline WGS using ImmuneLENS, a computational tool designed to quantify T-cell fractions from WGS data (16). Focusing on effector CD4^+^ and CD8^+^ α–β T cells from blood and lymph node, we observed a significant positive correlation between our cfDNA-derived T-cell ranks and the ImmuneLENS T-cell fraction (r = 0.18, *P* = 0.047; Fig. 2C), supporting the validity of the cfDNA-based cell type inference approach.

When repeating the analysis in the MIBC cohort, the most prominent shifts in cfDNA composition among patients with cancer compared with controls were an increase in lymphocyte-derived cfDNA and a decrease in monocyte-derived cfDNA, comparable with the patterns observed in the colorectal cancer cohort (Fig. 2D). However, no significant differences in monocyte or lymphocyte ranks were observed across ctDNA TF groups (Fig. 2E; Supplementary Fig. S3B). Furthermore, cfDNA-derived effector CD4^+^ and CD8^+^ α–β T-cell ranks from lymph node and blood showed a significant positive correlation with ImmuneLENS-estimated T-cell fractions (r = 0.24, *P* = 0.016; Fig. 2F).

We next explored cell type–specific alterations in cfDNA profiles by comparing cfDNA contributions from lymphocytes, granulocytes, and monocytes between pretreatment cancer samples and healthy controls. In the colorectal cancer cohort, 72 cell types were significantly overrepresented and 41 were underrepresented relative to healthy controls (Fig. 2G). In the MIBC cohort, 77 cell types were overrepresented and 48 were underrepresented relative to healthy controls (Fig. 2H). Across both cohorts, overrepresented cell types were primarily from the adaptive immune system, including plasma cells and T-cell subsets. In contrast, underrepresented cell types were mainly innate immune cells, with monocyte subsets exhibiting the largest negative FCs. These findings suggest a cancer-associated shift in the relative cfDNA contributions from innate to adaptive immune cells, reflecting changes in immune system activity and composition in the context of cancer.

### cfDNA-derived immune cell type signatures are predictive of cancer

To assess the diagnostic potential of cfDNA-derived immune signatures, we trained a classifier to distinguish pretreatment patients with cancer from healthy controls using cell type ranks from monocytes, lymphocytes, and granulocytes as input features. Given the relatively high dimensionality of the feature space (170 cell types) relative to the sample size, we used a LASSO-regularized logistic regression model for cancer classification (see “Materials and Methods”).

The model achieved an average area under the receiver operating characteristic curve (AUC) of 0.793 [95% confidence interval (CI), 0.716–0.870] for colorectal cancer and 0.745 (95% CI, 0.648–0.841) for MIBC using LOOCV (Fig. 2I). Notably, many patients across the cohorts had ctDNA TFs below 1% (Supplementary Fig. S2), making the observed sensitivity encouraging given that the method relies only on immune-derived cfDNA rather than tumor-specific signals.

To evaluate immune model predictions relative to ctDNA detection, we first compared model estimates between healthy controls and ctDNA TF groups. In both colorectal cancer and MIBC, prediction probabilities were significantly higher in patients with cancer compared with healthy controls (Supplementary Fig. S4A and S4B). However, no significant differences were observed between ctDNA TF groups, although an increasing trend with higher TF was observed in colorectal cancer. We next compared immune model detection rates with ctDNA detection using a 95% specificity threshold for immune model predictions. In colorectal cancer, the immune model detected 63% of ctDNA-positive samples and 38% of ctDNA-negative samples, whereas in MIBC, the detection rates were 46% and 35%, respectively (Supplementary Fig. S4C and S4D).

We further explored the prognostic value of immune cell type signatures for RFS. Immune model detection alone was not predictive of RFS in either colorectal cancer or MIBC (Supplementary Fig. S4E and S4F). Stratification by both immune score and ctDNA status revealed no difference in RFS among ctDNA-positive, ctDNA-negative, and ctDNA-negative/immune score–positive patients in the colorectal cancer cohort (Supplementary Fig. S4G). In contrast, a significant difference in RFS was observed across groups in the MIBC cohort; however, this was driven by poorer outcomes in the ctDNA-positive patients, whereas the ctDNA-negative/immune score–positive patients showed no separation from the ctDNA-negative patients (Supplementary Fig. S4H).

Together, these results indicate that immune cell ranks provide cancer-predictive information that is largely independent of tumor burden but with lower sensitivity and specificity than ctDNA and limited prognostic utility.

### cfDNA-derived immune cell type signatures change during therapy

Although treatment-naïve differences in cfDNA-derived cell type contributions may reflect underlying changes between patients with cancer and healthy individuals, their temporal dynamics during therapy remain less characterized. To investigate how the immune cell signatures evolve over the course of treatment, and how this may relate to clinical outcomes, we evaluated longitudinal plasma samples in the two cancer cohorts.

**Figure 3.**
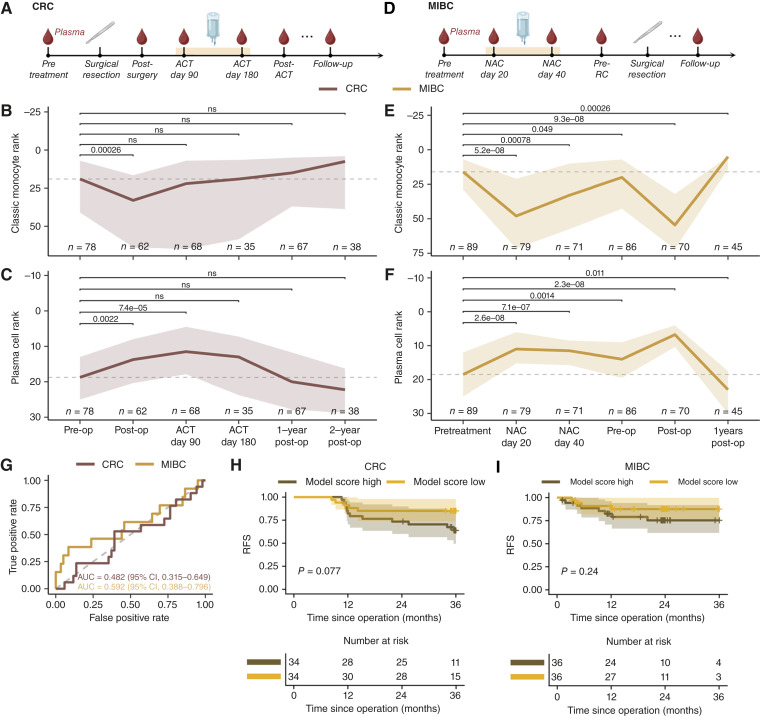
Longitudinal dynamics of cfDNA-derived immune cell contributions during therapy. **A,** Schematic of the treatment timeline for patients with colorectal cancer (CRC), including surgery and ACT. **B,** Longitudinal changes in cfDNA-derived rank of classic monocytes in patients with CRC. Number of patients per group is indicated. *P* values were calculated using two-sided Wilcoxon rank-sum tests comparing baseline (pre-op) to each time point. ns, not significant. **C,** Same as **B**, for plasma cell ranks in CRC. **D,** Schematic of the treatment timeline for patients with MIBC, including NAC, pre-RC, and follow-up time points. **E,** Longitudinal classic monocyte ranks in patients with MIBC, as in **B**. **F,** Same as **E**, for plasma cell ranks in MIBC. **G,** Receiver operating characteristic (ROC) curves for LASSO-regularized logistic regression models predicting relapse in CRC (red) and MIBC (yellow) based on cfDNA-derived immune features. Models were trained using the rank change between the baseline sample and the first chemotherapy time point (pre-op vs. ACT day 90 for CRC; pre-treat vs. NAC day 20 for MIBC) for the 10 cell types with highest IQR in rank change. Models were evaluated using LOOCV. Area under the curve (AUC) and 95% CIs are indicated. **H,** Kaplan–Meier curves of RFS for patients with CRC stratified into high (brown) and low (yellow) relapse probability groups based on the median predicted probability from the CRC model in **G**. Time is shown in months since operation. **I,** Same as **H**, for MIBC.

We first analyzed 348 longitudinal plasma samples collected from patients in the colorectal cancer cohort undergoing standard treatment, which included surgical resection of the tumor followed by ACT for a median of 155 days (IQR, 106–171 days; Fig. 3A). We examined cfDNA-derived ranks of classic monocytes from immune-related tissues (spleen, lymph node, blood, and thymus), which ranked among the most underrepresented cell types in pretreatment colorectal cancer samples compared with healthy controls (Fig. 2G). Monocyte ranks showed a modest decrease after surgery (*P* = 0.00026), with no significant changes during ACT or follow-up (Fig. 3B). In contrast, plasma cells—among the most overrepresented cell types in colorectal cancer relative to controls (Fig. 2G)—showed a significant increase following surgery and during ACT (post-op: *P* = 0.0022; ACT day 90: *P* = 7.4e−5; Fig. 3C), followed by a return to baseline at the 1-year follow-up.

We performed a similar analysis in the MIBC cohort, examining 440 longitudinal plasma samples from patients undergoing NAC for a median of 68.5 days (IQR: 61.2–78 days) followed by RC (Fig. 3D). For classic monocytes, we observed an initial decline in the cfDNA-derived rank during NAC (day 20: *P* = 5.2e−8; day 40: *P* = 0.00078; Fig. 3E), a return to baseline levels at the pre-op time point, and a further decrease following RC (*P* = 9.3e−8). In contrast, plasma cell–derived cfDNA displayed the opposite trajectory with an increase during NAC (day 20: *P* = 2.6e−8; day 40: *P* = 7.1e−7), a continued rise after RC (*P* = 2.3e−8), and a return to baseline 1 year post-RC (Fig. 3F).

Previous studies have demonstrated substantial interindividual variability in hematopoietic-derived measures (19). To better understand how this variability manifests in our cfDNA-derived immune cell profiles, we evaluated cell type rank trajectories at the individual patient level across treatment time points in the cancer cohorts. For high-abundance populations such as classic monocytes, treatment-induced changes were often sufficiently large to overcome background noise and thereby produce observable shifts in group-level means (Supplementary Fig. S5A and S5B). In contrast, lower-abundance populations like CD4^+^, α–β T cells exhibited more subtle changes that were frequently indistinguishable from interindividual variability (Supplementary Fig. S5C and S5D). In such contexts, comparing group-level means may fail to capture biologically relevant dynamics, particularly in smaller cohorts. These findings suggest that the absence of significant shifts in many cell types may reflect limitations in detecting subtle signals in the context of both technical noise and intrinsic variability in hematopoietic-derived cfDNA measures.

### cfDNA-derived immune cell type changes during chemotherapy are not predictive of patient outcome

We next evaluated whether changes in cfDNA-derived immune cell type composition during chemotherapy could predict patient relapse. For each patient, we calculated the change in cell type rank between the pretreatment sample (pre-op for colorectal cancer; pretreatment for MIBC) and the first chemotherapy time point (day 90 of ACT for colorectal cancer; day 20 of NAC for MIBC). To reduce dimensionality, we selected the top 10 immune cell types (monocytes, lymphocytes, and granulocytes) with the highest IQR in rank change. These features were used to train a LASSO-regularized logistic regression model to classify relapse.

The model demonstrated no predictive value, yielding AUCs of 0.482 (95% CI, 0.315–0.649) for colorectal cancer and 0.592 (95% CI, 0.388–0.796) for MIBC under LOOCV (Fig. 3G). Stratification of patients into high- and low-risk groups based on the median model-predicted relapse probability revealed no significant differences in RFS in either cohort, although we did observe a nonsignificant trend toward improved RFS in the low-risk group (Fig. 3H and I).

Collectively, these results suggest that cfDNA-derived immune cell type profiles may reflect treatment-induced changes in the immune cell composition. However, these are not consistently associated with patient relapse or survival.

## Discussion

In this study, we demonstrate that cfDNA coverage around TSSs reflects gene expression patterns in hematopoietic cells and can be integrated with single-cell transcriptomic references to infer the relative contributions of cell types to the cfDNA pool. This approach complements previous cfDNA deconvolution strategies based on DNA methylation (2), gene expression (9), or chromatin accessibility (20). However, it extends the field by focusing on systemic immune dynamics rather than tissue-specific signals, which are often sparse or undetectable in low-burden disease. Moreover, we analyze patients with earlier-stage cancer and incorporate longitudinal sampling to track potential immune cell changes as a response to treatment.

The cfDNA-derived cell type ranks recapitulated patterns previously observed using methylation-based (2) and window-protection score (9) deconvolution methods. Applying these ranks in a case–control analysis revealed that patients with cancer exhibit an increased cfDNA contribution from adaptive immune cell types—particularly T cells and plasma cells—alongside a relative depletion of innate immune cell types such as monocytes and granulocytes. These results suggest a systemic shift in immune cell turnover and activation associated with malignancy, consistent with earlier reports on cfDNA-derived cell type contributions in cancer (9, 10), though typically more pronounced in patients with advanced-stage disease. Cancer classification based on cfDNA-derived immune signatures yielded only modest accuracy and seemed less sensitive and specific than tumor-informed ctDNA-based detection. In addition, Kaplan–Meier analysis showed no association between immune model detection and RFS. This limited diagnostic and prognostic performance of the immune model is likely due to the low tumor burden in our cohorts, in which plasma ctDNA TFs were below 1% for many patients, which has also challenged prior tumor naïve classifiers based on similar approaches (9, 10, 15). Most fragmentomics studies focus on tissue-specific cancer signals such as gene expression (8) or transcription factor binding (15). In contrast, our approach captures systemic immune remodeling, which is inherently less specific to cancer but may still provide complementary information for noninvasive disease detection. In this way, our cfDNA-derived immune cell ranks may carry relevant information for cancer analysis; however, in the present form, the signals seem too noisy to provide reliable diagnostic or prognostic value. More accurate measures of immune cell abundance may enhance their clinical utility.

We observed no correlation between immune cell shifts and the ctDNA TF, except for a significant increase in lymphocyte-derived cfDNA with increasing ctDNA TF in the colorectal cancer cohort. The lack of association likely reflects both the high proportion of patients with low ctDNA tumor burden and the subtlety of immune-related cfDNA changes. Under healthy conditions, lymphocyte-derived cfDNA is estimated to constitute approximately 10% of the total cfDNA pool (2), suggesting that disease-related changes in this fraction may be too small to rise above the intrinsic background variability of cfDNA fragmentation. Thus, the absence of a strong association between cell type ranks and tumor burden may reflect the subtlety of the signal rather than the absence of a biological relevance.

In the longitudinal setting, we observed systemic shifts in cfDNA-derived cell type contributions following invasive treatments with chemotherapy and surgery. These changes were characterized by a decrease in classic monocyte-derived cfDNA and an increase in plasma cell–derived cfDNA. Such patterns may reflect widespread cell death in response to cytotoxic chemotherapy and surgical intervention or alterations in immune cell composition in response to treatment. Given the established role of the immune system in cancer progression and therapeutic response, we hypothesized that these cfDNA-derived cell type dynamics might be predictive of clinical outcome. However, changes in cell type contributions during chemotherapy were not predictive of relapse or clinical outcome. This lack of association can likely also be attributed to the subtle magnitude of changes in lower-abundance cell types, which are difficult to detect above the background fragmentation noise of cfDNA. Additionally, the high interindividual variability in hematologic measures, previously described by Foy and colleagues (19), likely contributes to the limited ability to detect changes in cfDNA-derived signals during treatment. To address this, we evaluated the cell type dynamics within individual patients across treatment time points, which confirmed that although some patients exhibited treatment-induced changes, these shifts were too subtle to overcome the high interpatient variability observed across the cohort.

Another key limitation when interpreting cfDNA-derived cell type ranks is that cfDNA originates from dying cells and therefore reflects cellular turnover rather than absolute circulating counts. Consequently, an apparent rise in a given cell type may arise from either greater abundance or increased turnover due to apoptosis. Fox-Fisher and colleagues (21) demonstrated that cfDNA better captures cellular turnover dynamics rather than blood cell counts, with release rates varying by cell lifespan, physiologic conditions, and disease status. They also reported substantial interindividual and intraindividual variability in cfDNA measurements, which further challenges detection of subtle longitudinal changes, consistent with our observations.

Therefore, to more accurately quantify these dynamics and assess their clinical relevance, more sensitive approaches may be required. For instance, cfDNA methylation–based deconvolution, as previously described by Moss and colleagues (2, 22), could improve resolution of cell type–specific contributions. Alternatively, longitudinal single-cell transcriptomic profiling of peripheral blood mononuclear cells may provide a more direct readout of immune remodeling during therapy and its relationship to treatment outcome.

In summary, we confirm and extend previous findings from cfDNA transcriptomic studies, demonstrating that coverage at TSSs can reliably infer gene expression of hematopoietic cells and enable deconvolution of cell types from plasma. We identify substantial shifts in cfDNA immune cell contributions between patients with cancer and healthy individuals, characterized by elevated signals from adaptive immune cells and a reduction in innate immune cell–derived cfDNA. These immune signatures evolve dynamically during chemotherapy and surgical treatment; however, they do not distinguish between patients who relapse and those who remain disease-free. Together, our findings highlight the potential of cfDNA profiling as a noninvasive tool for monitoring systemic immune responses in cancer, offering a complementary perspective to tumor-derived ctDNA analyses.

## Supplementary Material

Supplementary Figure 1 Supplementary Figure 1 | Cell type contribution to compartments grouped by tissue. Cell type ranks of cell types from the compartments endothelial, epithelial, germline, and stromal grouped by tissue in healthy individuals (n=30). This is defined from the Tabula Sapiens definition of cell types, which can originate from more than one tissue.

Supplementary Figure 2 Supplementary Figure 2 | ctDNA-based analysis of the cancer cohorts. A. ctDNA detection status in the colorectal cancer (CRC) and muscle invasive bladder cancer (MIBC) cohorts. ND = not detected, D = detected, NA = not available. Number and % of patients in each group are indicated. B. Plasma ctDNA tumor fractions (%) in the ctDNA-positive subgroup of the CRC (red) and MIBC (yellow) cohorts. Dashed line indicates ctDNA TF = 1%.

Supplementary Figure 3 Supplementary Figure 3 | Comparison of cell type ranks to plasma ctDNA tumor fraction. A. Cell type ranks of monocytes and lymphocytes from spleen, lymph node, blood, and thymus compared to the plasma ctDNA tumor fraction (TF) in colorectal cancer (CRC). Patients are stratified by ctDNA status into: not detected (ND), TF < 0.1%, 0.1% < TF < 1%, and TF > 1%. P-values were calculated using two-sided Wilcoxon rank-sum tests. B. Same as A for muscle invasive bladder cancer (MIBC).

Supplementary Figure 4 Supplementary Figure 4 | Comparison of immune model detection of cancer to ctDNA-based detection. A. Immune model prediction probability of patient relapse in colorectal cancer (CRC) compared across healthy individuals and ctDNA tumor fraction (TF) groups: not detected (ND), TF < 1%, TF > 1%. P-values were calculated using two-sided Wilcoxon rank-sum tests. B. Same as A for muscle invasive bladder cancer (MIBC). C. Comparison of ctDNA-based cancer detection and immune model detection in CRC. The number of patients detected (red) and not detected (grey) with the immune model among ctDNA-positive and ctDNA-negative patients are indicated. P-values calculated using Fisher's exact test. D. Same as C for MIBC. E. Kaplan–Meier curves showing 3-year recurrence-free survival (RFS) in patients with CRC, stratified by immune model detection: yes (red), blue (no). F. Same as E for MIBC. G. Same as E, but patients are stratified into three groups: ctDNA-negative (ctDNA–; grey), ctDNA-negative/Immune score-positive (ctDNA–/Immune score+; blue), and ctDNA-positive (ctDNA+; red). Immune score positivity was defined using a 95% specificity threshold from the LASSO model described in Figure 2I. RFS was calculated from the date of surgery. Shaded areas represent 95% confidence intervals. H. Same as G, for patients with MIBC.

Supplementary Figure 5 Supplementary Figure 5 | Longitudinal dynamics of cfDNA-derived immune cell type ranks for individual patients. A. Longitudinal trajectories of classical monocyte ranks in patients with colorectal cancer (CRC) across treatment. Grey lines represent individual patients, the red line highlights a selected patient. Blue bar indicates the pre-treatment median ± interquartile range (IQR) or zero when IQR < 0. B. Same as A, for patients with muscle-invasive bladder cancer (MIBC). C. Same as A, showing CD4+, α-β T cells ranks in CRC. D. Same as C, for MIBC. ACT = adjuvant chemotherapy. NAC = neoadjuvant chemotherapy. pre-treat = pre-treatment. pre-op = pre-operatively. pre-RC = pre-radical cystectomy. post-op = post-operatively. y = year.

Supplementary Table 1 Supplementary Table 1 | Definition of cell type groupings of the tabula sapiens reference dataset. Demonstrates cell type from the tabula sapiens reference dataset and the corresponding cell type category used.

## Data Availability

The analysis code used for carrying out and visualizing analyses is available from the corresponding author upon request. All data analyzed in this study were obtained from previously published studies [Frydendahl and colleagues (11) and Nordentoft and colleagues (12)]. Access to the underlying raw data should be requested from the corresponding authors of the original publications, where it is available upon reasonable request in accordance with their data sharing policies.
