# Supplementary figures and images for: Cell-Free DNA–Derived Immune Cell Ratios Uncover Cancer-Associated Systemic Changes
Source: Cancer Res Commun. 2026 Apr 13;6(4):811–20. doi: 10.1158/2767-9764.CRC-25-0747 (PMC13075467; doi:10.1158/2767-9764.CRC-25-0747)

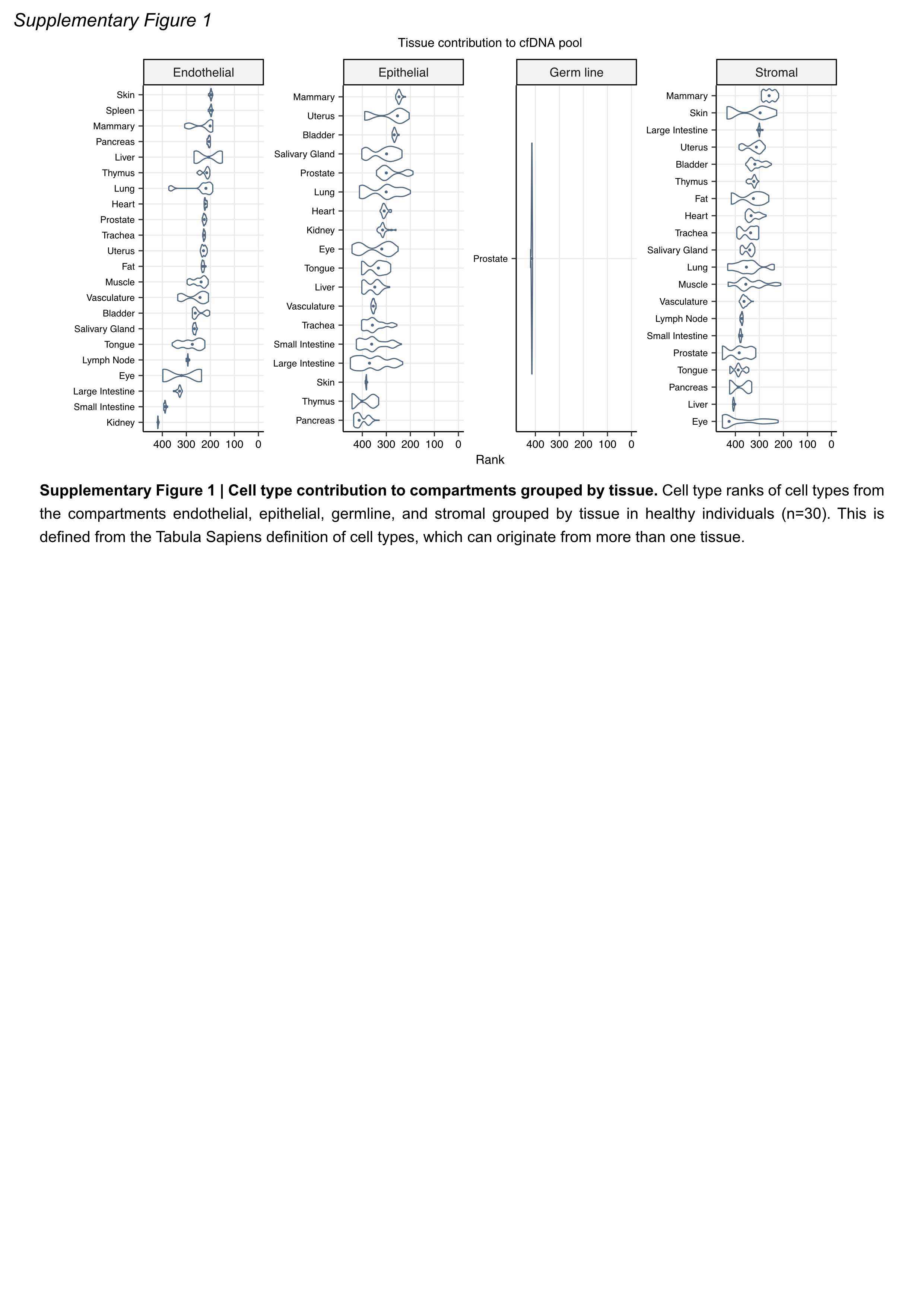

Supplement: Supplementary Figure 1 — | Cell type contribution to compartments grouped by tissue. Cell type ranks of cell types from the compartments endothelial, epithelial, germline, and stromal grouped by tissue in healthy individuals (n=30). This is defined from the Tabula Sapiens definition of cell types, which can originate from more than one tissue. [file crc-25-0747_supplementary_figure_1_suppsf1.png]

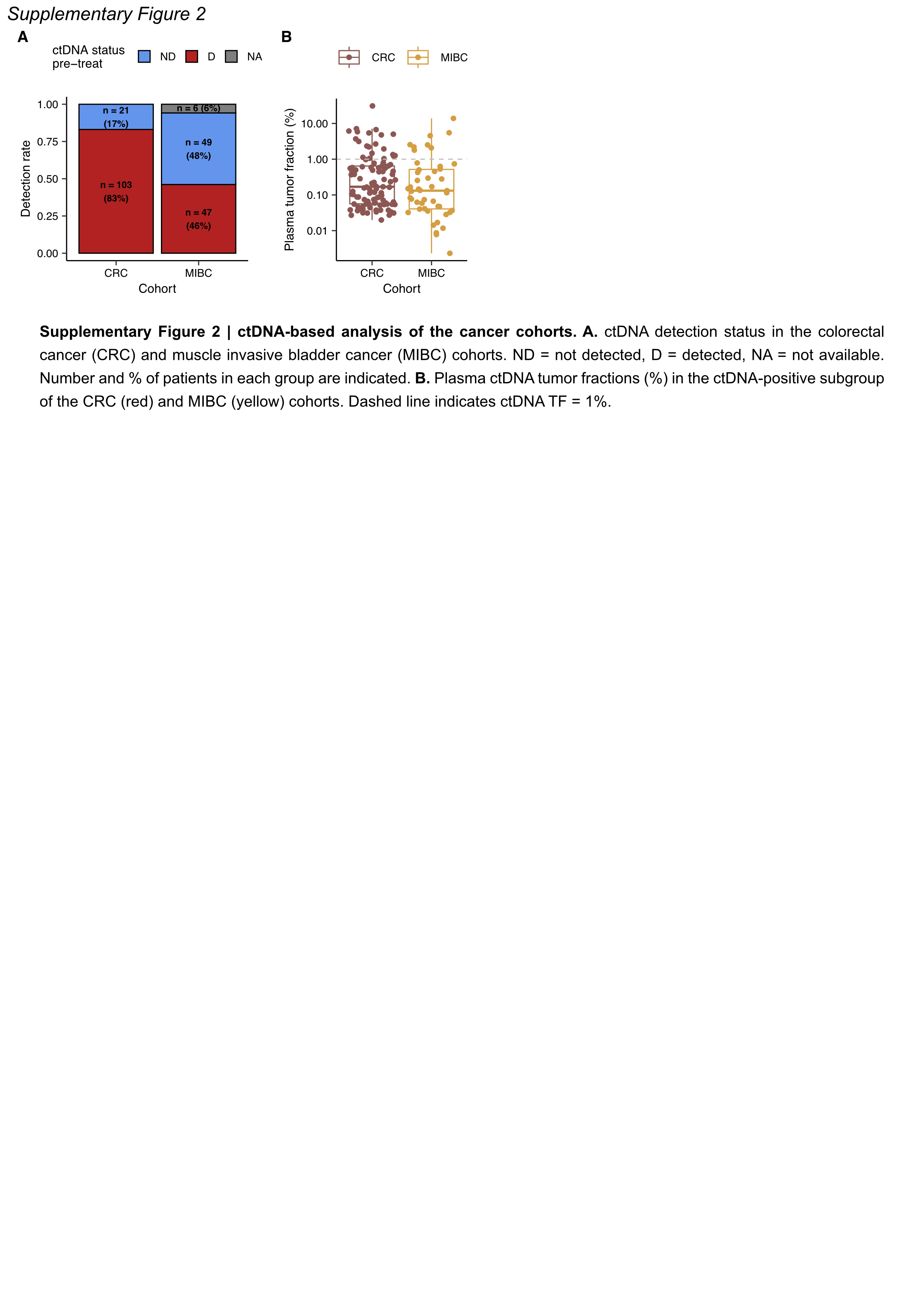

Supplement: Supplementary Figure 2 — | ctDNA-based analysis of the cancer cohorts. A. ctDNA detection status in the colorectal cancer (CRC) and muscle invasive bladder cancer (MIBC) cohorts. ND = not detected, D = detected, NA = not available. Number and % of patients in each group are indicated. B. Plasma ctDNA tumor fractions (%) in the ctDNA-positive subgroup of the CRC (red) and MIBC (yellow) cohorts. Dashed line indicates ctDNA TF = 1%. [file crc-25-0747_supplementary_figure_2_suppsf2.png]

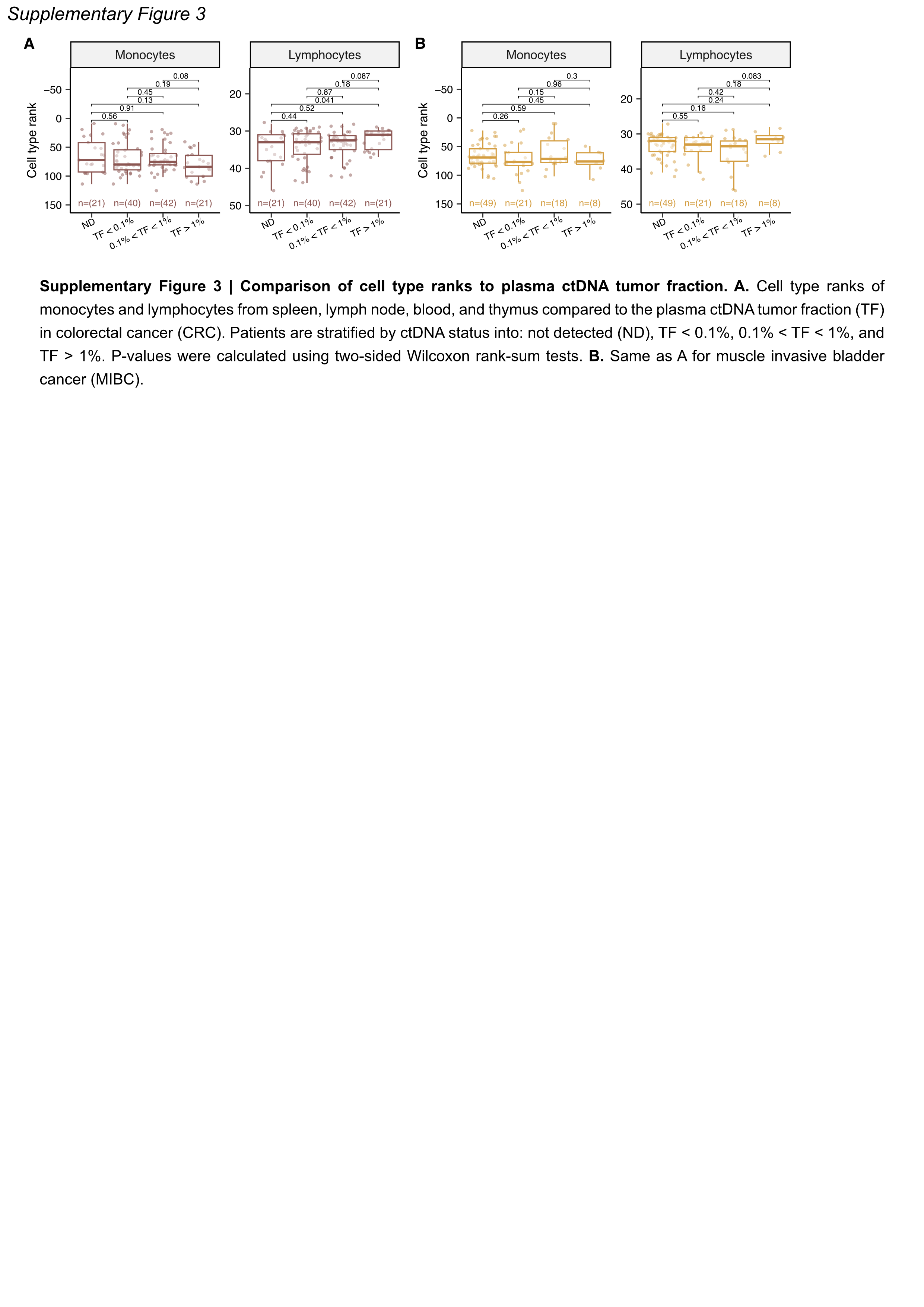

Supplement: Supplementary Figure 3 — | Comparison of cell type ranks to plasma ctDNA tumor fraction. A. Cell type ranks of monocytes and lymphocytes from spleen, lymph node, blood, and thymus compared to the plasma ctDNA tumor fraction (TF) in colorectal cancer (CRC). Patients are stratified by ctDNA status into: not detected (ND), TF < 0.1%, 0.1% < TF < 1%, and TF > 1%. P-values were calculated using two-sided Wilcoxon rank-sum tests. B. Same as A for muscle invasive bladder cancer (MIBC). [file crc-25-0747_supplementary_figure_3_suppsf3.png]

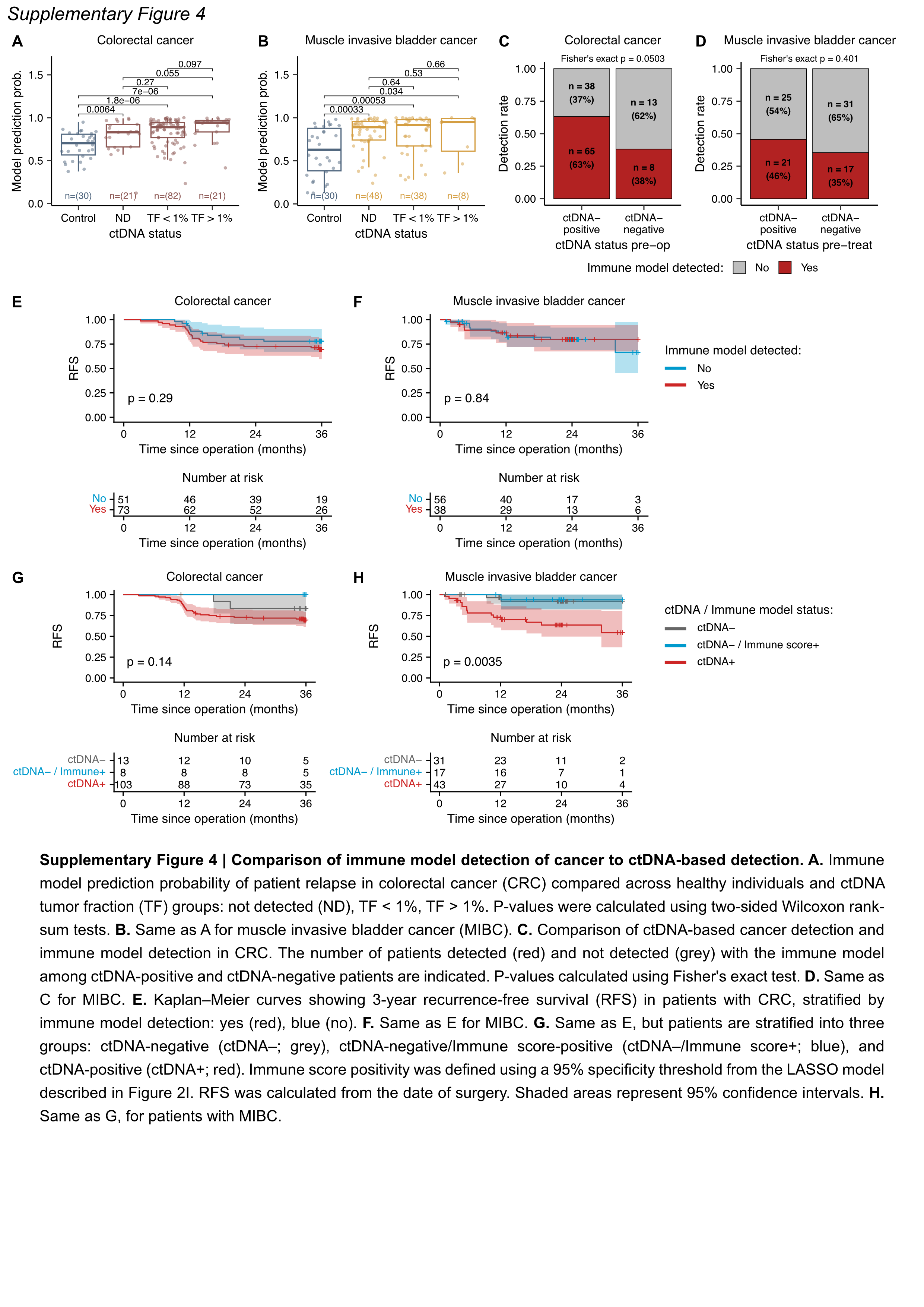

Supplement: Supplementary Figure 4 — | Comparison of immune model detection of cancer to ctDNA-based detection. A. Immune model prediction probability of patient relapse in colorectal cancer (CRC) compared across healthy individuals and ctDNA tumor fraction (TF) groups: not detected (ND), TF < 1%, TF > 1%. P-values were calculated using two-sided Wilcoxon rank-sum tests. B. Same as A for muscle invasive bladder cancer (MIBC). C. Comparison of ctDNA-based cancer detection and immune model detection in CRC. The number of patients detected (red) and not detected (grey) with the immune model among ctDNA-positive and ctDNA-negative patients are indicated. P-values calculated using Fisher's exact test. D. Same as C for MIBC. E. Kaplan–Meier curves showing 3-year recurrence-free survival (RFS) in patients with CRC, stratified by immune model detection: yes (red), blue (no). F. Same as E for MIBC. G. Same as E, but patients are stratified into three groups: ctDNA-negative (ctDNA–; grey), ctDNA-negative/Immune score-positive (ctDNA–/Immune score+; blue), and ctDNA-positive (ctDNA+; red). Immune score positivity was defined using a 95% specificity threshold from the LASSO model described in Figure 2I. RFS was calculated from the date of surgery. Shaded areas represent 95% confidence intervals. H. Same as G, for patients with MIBC. [file crc-25-0747_supplementary_figure_4_suppsf4.png]

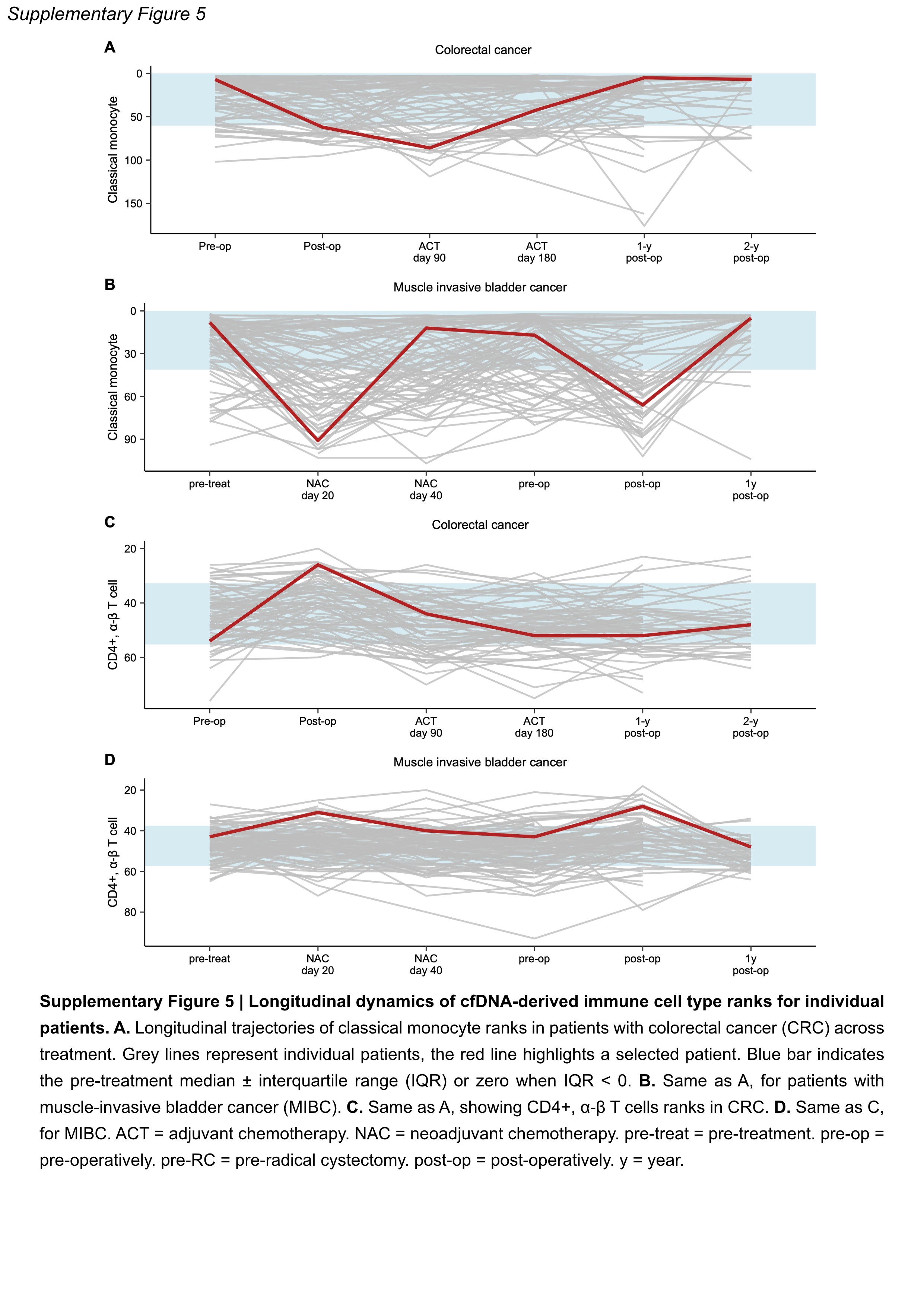

Supplement: Supplementary Figure 5 — | Longitudinal dynamics of cfDNA-derived immune cell type ranks for individual patients. A. Longitudinal trajectories of classical monocyte ranks in patients with colorectal cancer (CRC) across treatment. Grey lines represent individual patients, the red line highlights a selected patient. Blue bar indicates the pre-treatment median ± interquartile range (IQR) or zero when IQR < 0. B. Same as A, for patients with muscle-invasive bladder cancer (MIBC). C. Same as A, showing CD4+, α-β T cells ranks in CRC. D. Same as C, for MIBC. ACT = adjuvant chemotherapy. NAC = neoadjuvant chemotherapy. pre-treat = pre-treatment. pre-op = pre-operatively. pre-RC = pre-radical cystectomy. post-op = post-operatively. y = year. [file crc-25-0747_supplementary_figure_5_suppsf5.png]
